# Characteristics of *Enterococcus species* bloodstream infections among adults with and without onco-hematological malignancies: Experiences from the national center of Hungary

**DOI:** 10.1556/1886.2024.00011

**Published:** 2024-03-27

**Authors:** Bence Marosi, Béla Kádár, Anna Bruzsa, Laura Kocsis, Katalin Kamotsay, János Sinkó, Bálint Gergely Szabó, Botond Lakatos

**Affiliations:** 1 South Pest Central Hospital, National Institute of Haematology and Infectious Diseases, Albert Flórián Street 5-7., H-1097, Budapest, Hungary; 2 School of PhD Studies, Semmelweis University, Üllői Street 26., H-1085, Budapest, Hungary; 3 Departmental Group of Infectious Diseases, Department of Internal Medicine and Haematology, Semmelweis University, Albert Flórián Street 5-7., H-1097, Budapest, Hungary

**Keywords:** Enterococcus faecalis, Enterococcus faecium, Enterococcus sp., enterococcaemia, bloodstream infection, survival, mortality

## Abstract

**Introduction:**

Over the past decade, enterococcal bloodstream infection (BSI) shows increasing incidence globally among the elderly and in patients with comorbidities. In this study, we aimed to assess microbiological and clinical characteristics and long-term outcomes of BSIs caused by *Enterococcus* spp. in adult patients with and without active onco-hematological malignancies hospitalized at a national referral institute.

**Methods:**

A prospective analysis of consecutive enterococcal BSI cases was conducted in the National Institute of Hematology and Infectious Diseases (Budapest, Hungary) between December 2019 and April 2022. We compared characteristics and outcomes at 30-days and 1 year after diagnosis among patients with and without onco-hematological malignancies.

**Results:**

In total, 141 patients were included (median age 68 ± 21 years, female sex 36.9%), 37% (52/141) had active onco-hematological malignancies. The distribution of species was as follows: 50.4% *Enterococcus faecalis*, 46.1% *Enterococcus faecium*, 1.4% *Enterococcus avium* and *Enterococcus gallinarum*, and 0.7% *Enterococcus raffinosus*. No statistically significant differences in all-cause mortality rates were observed between patient subgroups at 30 days (32.7 vs. 28.1%; *P* = 0.57) and 1 year (75.0 vs. 60.7%; *P* = 0.09).

**Conclusion:**

Enterococcal bloodstream infections yielded a relevant burden of morbidity, but with no statistical difference in long-term outcomes of adult patients with and without active onco-hematological malignancies.

## Introduction

A bloodstream infection (BSI) constitutes a clinical phenomenon characterized by alterations in clinical, hemodynamic, and biochemical parameters, concomitant with the presence of pathogenic bacteria and/or fungi in the blood [1, 2]. Signifying a substantial healthcare burden, BSI accounts for approximately 2 million cases in adults, contributing to an estimated 250,000 annual fatalities in North America and Europe [3, 4]. The epidemiological landscape of BSI has undergone notable transformations in recent decades, owing to the advancing age of affected individuals and the integration of biotechnological innovations of medicine, particularly within the fields of oncology and hematology. In addition to BSI cases attributable to Gram-negative bacteria and fungi, Gram-positive pathogens, such as coagulase-negative staphylococci, enterococci, and *Staphylococcus aureus*, are assumed to have a prominent role in nosocomial infections [3, 5].

Enterococci, ranking as the second most prevalent causative agents of bloodstream infections due to Gram-positive bacteria in both Europe and the United States of America, exhibit an associated in-hospital mortality rate of up to 68% [6]. The management of enterococcaemia poses significant challenges, given the susceptibilities and acquired antibiotic resistances observed in certain enterococal species, often necessitating prolonged or combination antibiotic regimens, thereby indirectly contributing to hospitalization and heightened healthcare costs [7–10]. Notably, enterococci feature prominently as causative agents of infective endocarditis, constituting the third most prevalent pathogen group in high-income countries, where advanced age and complex comorbidities serve as established risk factors for this type of infection [11, 12]. Prompt recognition of enterococcaemia and the administration of appropriate antimicrobials are assumed to be of critical significance, as delayed or inadequate therapy correlates with adverse outcomes and increased mortality rates. A recent study has identified inadequate empirical antibiotic therapy in BSIs caused by *Enterococcus* spp. as an independent predictor of 30-day all-cause mortality, particularly among elderly patients [12].

In light of the existing literature, a preference for additional evidence is lacking, particularly concerning the follow-up of BSIs attributable to *Enterococcus* spp., especially within the context of Hungary. Therefore the present study aims to assess the microbiological and clinical characteristics, as well as long-term outcomes, of bloodstream infections caused by *Enterococcus* species among adult patients, with a specific focus on those with active onco-hematological malignancies.

## Methods

### Study design and settings

We conducted a prospective observational cohort study by enrolling consecutive adult patients admitted to South Pest Central Hospital, National Institute of Hematology and Infectious Diseases (Budapest, Hungary), during the period between December 2019 and April 2022. The study center operates within a national enrollment area with >500 beds.

### Patient identification and selection

Throughout the study duration, all hospitalized patients were considered potentially eligible for inclusion if a bloodstream infection caused by *Enterococcus* spp. was microbiologically confirmed during their hospitalization. For inclusion, a query from the electronic archiving system of our microbiology laboratory for *E.* spp. isolated from blood cultures during the study period was performed. The definition of a bloodstream infection required the isolation of *E.* spp. from at least one blood culture bottle aligning with a clinically congruent presentation. Pre-specified exclusion criteria comprised of: 1) unavailability of patient records in the hospital electronic filing system, 2) incongruence of the clinical case review with a bloodstream infection caused by *E.* spp., or 3) the microbiological specimen was gathered at a different hospital. All microbiological analyses were conducted at the Core Microbiology Laboratory of our institution, employing standard culture methods with Columbia +5% sheep blood agar (bioMérieux, Budapest Hungary), Chocolate PolyViteX agar (bioMérieux, Budapest Hungary) and CHROMID vancomycin-resistant enterococci (VRE) agar (bioMérieux, Budapest Hungary) for bacterial isolation, and VITEK-MS (bioMérieux, Budapest Hungary) for identification. For *in vitro* antibiotic susceptibility testing, we adhered to the current European Committee on Antimicrobial Susceptibility Testing (EUCAST) recommendations by using Mueller Hinton E agar (bioMérieux, Budapest Hungary), Kirby-Bauer disc diffusion (Oxoid, Basingstoke United Kingdom) and VITEK-2 Compact dilution methods (bioMérieux, Budapest Hungary).

### Data collection

Relevant data from the included cases were entered into an electronic data collection chart in an anonymized manner, by using a case report format standardized by the study group. The entirety of the hospitalization period was tracked through the electronic archiving system, while post-discharge long-term follow-up extended until patient demise or the last available outpatient/hospital care, utilizing data sourced from the social security database (National eHealth Infrastructure) of Hungary. The collected parameters encompassed: 1) age at diagnosis, gender; 2) comorbidities (essential hypertension, chronic heart, lung, kidney, liver, and cerebrovascular diseases, diabetes mellitus, active onco-hematologic malignancy, systemic autoimmune disease, systemic glucocorticosteroid use, tobacco and alcohol abuse) and Charlson index; 3) presence of intravascular/intracardial devices or artificial valves; 4) identified pathogens; 5) clinical outcomes; 6) long-term survival. Stratification of included patients into subcohorts was based on the presence or absence of active onco-hematological malignancy.

### Clinical and microbiological outcomes

The primary and secondary clinical outcomes were defined as 30-day and 1-year all-cause mortality after BSI diagnosis, respectively. Microbiological outcomes encompassed the species distribution and *in vitro* antibiotic susceptibility patterns of *Enterococcus* spp. isolated from blood cultures.

### Statistical analysis

Continuous variables are presented as median ± interquartile range (IQR) with minimum–maximum values, while categorical variables are expressed as absolute values (*n*) and relative proportions (%). The Mann-Whitney U-test or Fisher's exact test were performed for statistical comparisons, dependent upon variable distribution. Normality was assessed using the Shapiro-Wilk test. A two-sided *P*-value of <0.05 was considered statistically significant for all tests. Statistical analyses were conducted using MedCalc 22, with data collection executed through Microsoft Office Excel 2016.

### Ethics

Approval for the study protocol was obtained from the Institutional Ethics Committee of South Pest Central Hospital, National Institute of Hematology and Infectious Diseases (IKEB 37/2016). Given the nature of this study, informed consent from participants was deemed unnecessary. The study personnel adhered to the principles of the Declaration of Helsinki.

## Results

### Demographic and comorbidity characteristics

During the study period, 158 cases were investigated, of which 141 (89.2%) met study inclusion criteria. The demographic and comorbidity characteristics of the enrolled patients are presented in Table 1. The median age within the cohort was 68 ± 21 years, and 36.9% (52/141) of the patients were female. 37% (52/141) had active onco-hematological malignancies. The median Charlson index was 5 ± 4, with essential hypertension (59.6%, 84/141) and chronic cardiovascular diseases (33.3%, 47/141) emerging as the predominant comorbidities. Significant differences were only observed in the use of systemic use of glucocorticoids (0 vs. 3 *P* = 0.04) and in the incidence of chronic cerebrovascular disease (19 vs. 3 *P* = 0.01) between the groups. A minority of patients (2.1%, 3/141) had intracardiac devices, artificial valves were present in 2.8% (4/141) of cases. Transthoracic and transoesophageal echocardiography were conducted in 16.3% (23/141) and 0.7% (1/141) of cases during hospitalization, respectively, resulting in a solitary case with the final diagnosis for infective endocarditis. The median duration of hospital stay was 28 ± 28.0 days.

**Table 1.** Demographic and clinical parameters of adult patients with and without onco-hematological malignancies diagnosed with *Enterococcus* spp. bloodstream infection

Parameter	Total (*n* = 141)	Non-onco-hematological (*n* = 89)	Onco-hematological (*n* = 52)	*P* value
**Age** (year, median ± IQR, min–max)	68.0 ± 21 (22–101)	70.0 ± 20 (28–90)	64.5 ± 21 (23–101)	0.01
**Female sex** (*n*, %)	52 (36.9)	33 (38.4)	19 (52.8)	1.0
**Comorbidities** (*n*, %)				
- Essential hypertension	84 (59.6)	57 (64.0)	27 (51.9)	0.21
- Chronic heart disease	47 (33.3)	35 (39.3)	12 (23.1)	0.06
- Chronic vascular disease	27 (19.1)	20 (22.5)	7 (13.5)	0.26
- Chronic lung disease	18 (12.8)	13 (14.6)	5 (9.6)	0.44
- Chronic kidney disease	12 (8.5)	10 (11.2)	2 (3.8)	0.21
- Chronic liver disease	6 (4.3)	6 (6.7)	0 (0.0)	0.08
- Chronic cerebrovascular disease	22 (15.6)	19 (21.3)	3 (5.8)	0.01
- Diabetes mellitus	36 (25.5)	27 (30.3)	9 (17.3)	0.11
- Active oncological malignancy	17 (12.1)	0 (0.0)	17 (12.1)	n.a.
- Active hematological malignancy	36 (25.5)	0 (0.0)	36 (25.5)	n.a.
- Systemic autoimmune disease	1 (0.7)	0 (0.0)	1 (0.7)	0.36
- Systemic use of glucocorticosteroids	3 (2.1)	0 (0.0)	3 (2.1)	0.04
- Alcohol abuse	13 (9.2)	9 (10.1)	4 (7.7)	0.76
- Tobacco use	5 (3.5)	3 (3.4)	2 (3.8)	1.00
**Charlson index** (median ± IQR, min–max)	5.0 ± 4 (0–11)	5.0 ± 4 (0–10)	6.0 ± 4 (2–11)	0.17
**Number of comorbidities per person** (median ± IQR, min–max)	3.1 ± 3 (0–8)	3.0 ± 3 (0–7)	3.0 ± 3 (1–8)	0.06
**Hospital length of stay** (day, median ± IQR, min–max)	28 ± 28 (1–198)	26 ± 28 (1–88)	29 ± 34 (1–198)	0.36
**Number of patients admitted to the ICU** (*n*, %)	77 (54.6)	52 (58.4)	25 (69.4)	0.29
**30-day all-cause mortality** (*n*, %)	42 (29.7)	25 (28.1)	17 (32.7)	0.57
**1-year all-cause mortality** (*n*, %)	93 (66.0)	54 (60.7)	39 (75.0)	0.09

ICU: intensive care unit, IQR: interquartile range, n.a.: not applicable.

### Clinical outcomes

**Fig. 1.**
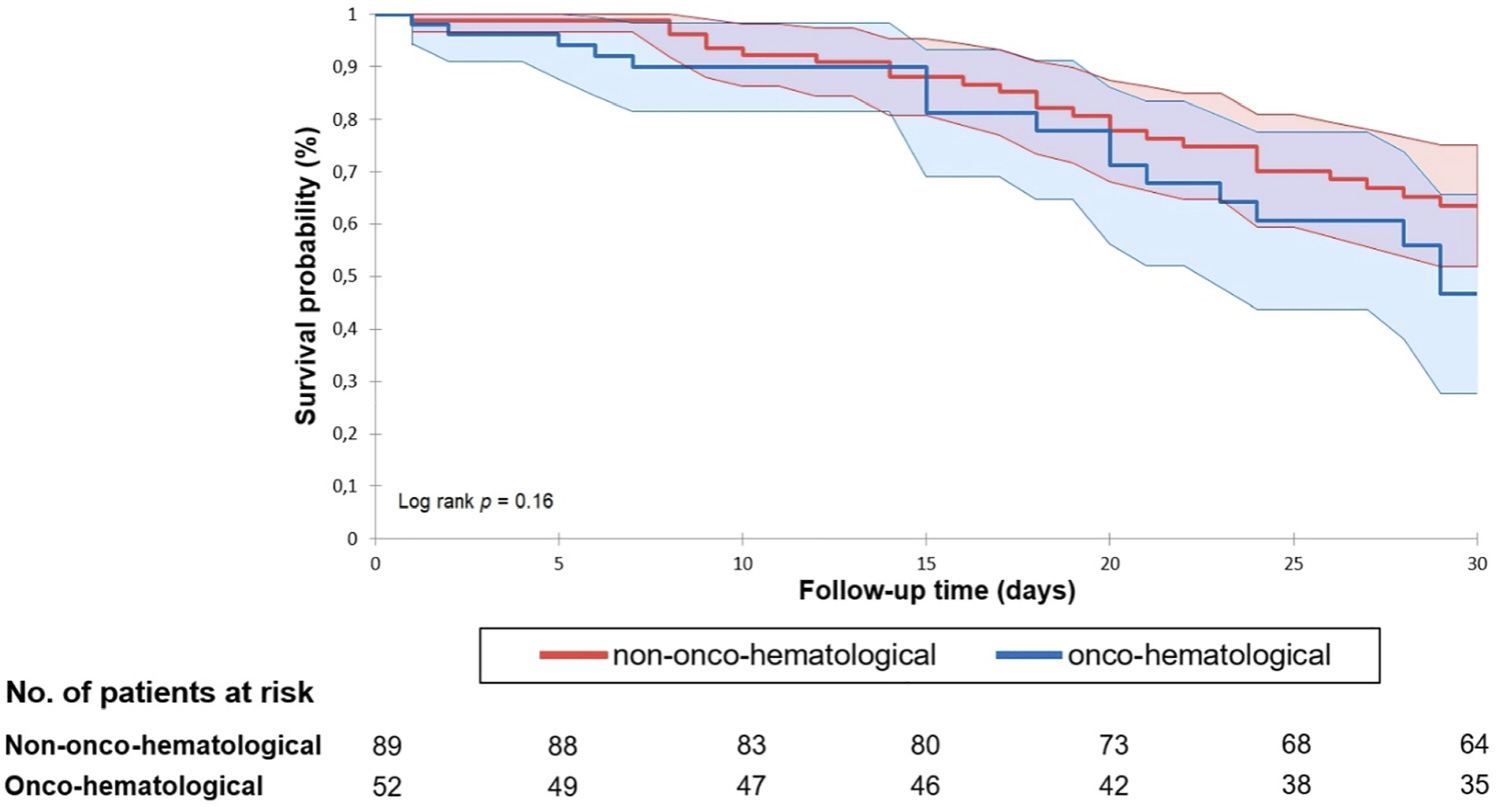
30-day Kaplan–Meier survival distributions with numbers of at-risk patients with and without onco-hematological malignancies diagnosed with *Enterococcus* spp. bloodstream infection. Survival curves (thick lines) are depicted along with their corresponding 95% confidence intervals (colored regions outlined by thin lines)

**Fig. 2.**
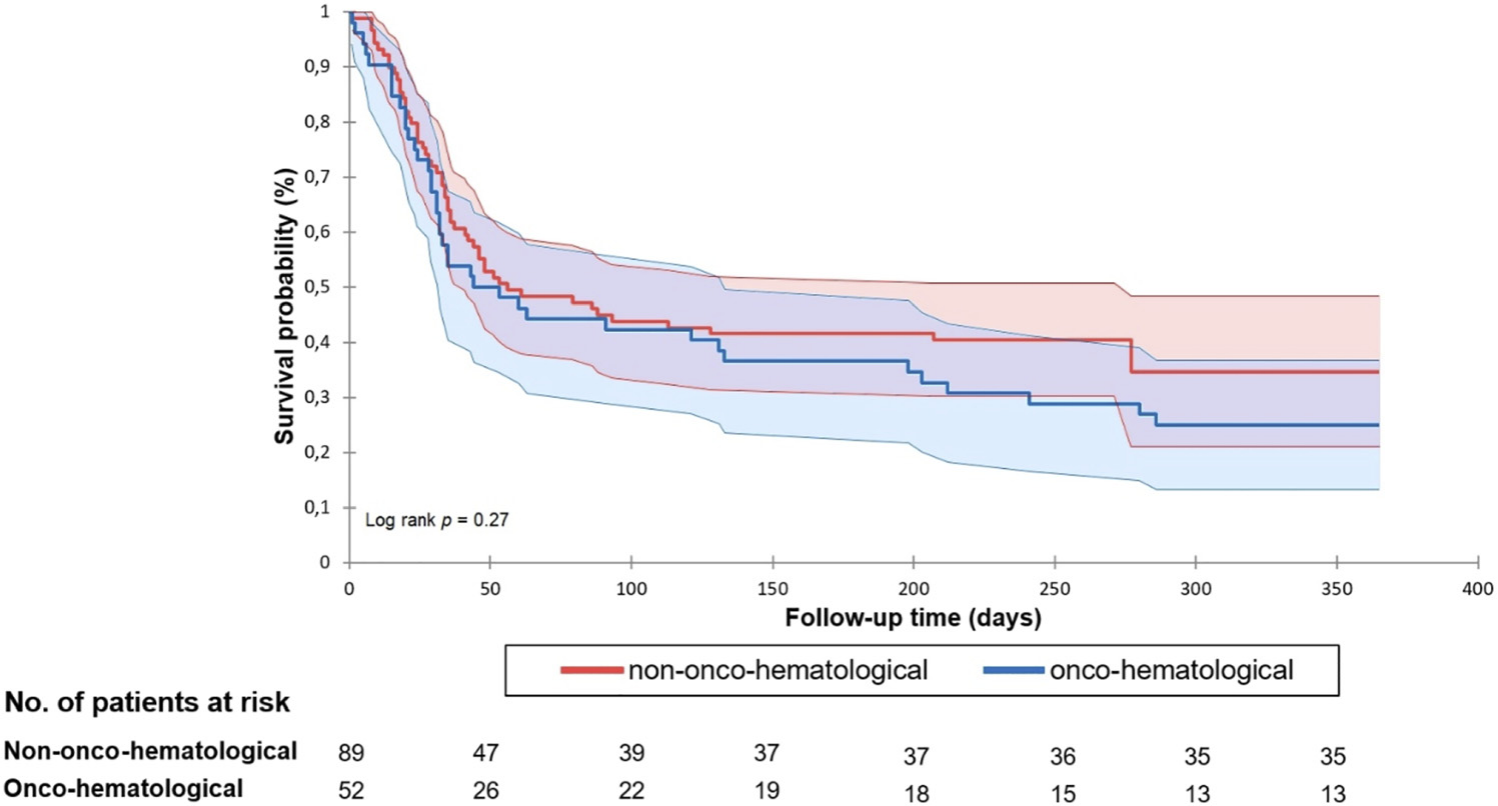
1-year Kaplan–Meier survival distributions with numbers of at-risk patients with and without onco-hematological malignancies diagnosed with *Enterococcus* spp. bloodstream infection. Survival curves (thick lines) are depicted along with their corresponding 95% confidence intervals (colored regions outlined by thin lines)

The 30-day all-cause mortality rate was 29.8% (42/141), as outlined in Table 1. Among the deceased, 53.8% (50/93) were males, 40.5% (17/42) had active onco-hematological disease, and a substantial majority (79.2%, 33/42) died during intensive care unit (ICU) treatment. Community-acquired enterococcemia accounted for 4.7% (2/42) of the reported deaths. Only one case without any documented chronic disease ended in death. There was no significant statistical difference in all-cause mortality between the groups. The median follow-up duration extended to 300 ± 23 days, with 34.0% (48/141) of patients surviving at the end of the follow-up period. Of the total deaths, 45.2% (42/93) occurred within 30 days, with the subsequent distribution of 40.9% (38/93) within 2 months, 7.5% (7/93) within 6 months, and 6.5% (6/93) within one year. Kaplan-Meier analysis of the 30-day and 1-year survival distributions did not reveal statistically significant distinctions between the two subcohorts (please refer to Figs 1 and 2).

### Microbiological characteristics

The distribution of *E.* spp. isolates and their *in vitro* antibiotic susceptibility profiles are depicted in Table 2. Species distribution was led by *Enterococcus faecalis* (50.4% of all isolates), followed by *Enterococcus faecium* (46.1%), *Enterococcus avium, Enterococcus gallinarum* and *Enterococcus raffinosus* (1.4, 1.4, and 0.7%), respectively. *E. faecalis* isolates showed susceptibility to gentamicin in 59.2%, while susceptibility rates of *E. faecium* were lower, with 9.2, 50.8, 52.3 and 58.5% to ampicillin, teicoplanin, vancomycin and gentamicin, respectively. Other enterococcal isolates showed species specific resistance profiles. Polymicrobial bloodstream infections involving bacteria other than enterococci were documented in 26.9% (38/141) of cases. Notably, 54.8% of all cases were of nosocomial origin. Four out of five patients with non-faecalis and non-faecium enterococcal strains had an underlying immuncompromising condition. Demographic and clinical parameters of adult patients with *E. faecalis* and *E. faecium* bloodstream infections are depicted in Table 3. Significant differences were only observed in hematological malignancies (10 vs. 26 *P* = 0.001) and chronic cerebrovascular diseases (17 vs. 4 *P* = 0.04).

**Table 2.** Results of *in vitro* antibiotic susceptibility testings of *Enterococcus* sp. strains isolated from blood cultures of adult patients with and without onco-hematological malignancies

Species	Number of isolates (*n*)	Number of isolates showing in vitro susceptibility to the tested antibiotics (*n*, %)*							
		*Ampicillin*	*Gentamicin*	*Imipenem/cilastatin*	*Linezolid*	*Piperacillin/tazobactam*	*Teicoplanin*	*Tigecycline*	*Vancomycin*
*E. faecalis*	71	71 (100)	42 (59.2)	71 (100)	71 (100)	71 (100)	71 (100)	71 (100)	71 (100)
*E. faecium*	65	6 (9.2)	38 (58.5)	6 (9.2)	65 (100)	6 (9.2)	33 (50.8)	65 (100)	34 (52.3)
*E. avium*	2	2 (100)	0	2 (100)	n.d.	2 (100)	n.d.	n.d.	2 (100)
*E. gallinarum*	2	2 (100)	2 (100)	1 (50)	2 (100)	1 (50)	2 (100)	2 (100)	0
*E. raffinosus*	1	0	1 (100)	0	1 (100)	0	0	1 (100)	0
TOTAL	141	81 (57.5)	83 (58.9)	80 (56.7)	141 (100)	80 (56.7)	106 (78.7)	141 (100)	107 (75.9)

* Percentages are reported in proportion to the number of isolates of the given species.

n.d.: no data.

**Table 3.** Demographic and clinical parameters of adult patients with *Enterococcus faecalis* and *Enterococcus faecium* bloodstream infections

Parameter	*E. faecalis* (*n* = 71)	*E. faecium* (*n* = 52)	*P* value
**Age** (year, median ± IQR, min–max)	70.0 ± 15 (28–90)	64.0 ± 24 (23–93)	0.08
**Female sex** (*n*, %)	27 (38.0)	23 (35.4)	0.75
**Comorbidities** (*n*, %)			
- Essential hypertension	46 (64.8)	35 (53.8)	0.19
- Chronic heart disease	27 (38.0)	20 (30.8)	0.37
- Chronic vascular disease	18 (25.4)	9 (13.8)	0.09
- Chronic lung disease	9 (12.7)	9 (13.8)	0.84
- Chronic kidney disease	8 (11.3)	3 (4.6)	0.16
- Chronic liver disease	4 (5.6)	1 (1.5)	0.21
- Chronic cerebrovascular disease	17 (23.9)	4 (6.2)	0.04
- Diabetes mellitus	20 (28.2)	16 (24.6)	0.63
- Active oncological malignancy	8 (11.3)	7 (10.8)	0.93
- Active hematological malignancy	10 (14.1)	26 (40.0)	0.001
- Systemic autoimmune disease	1 (1.4)	0 (0.0)	0.95
- Systemic use of glucocorticosteroids	0 (0.0)	3 (4.6)	0.27
- Alcohol abuse	8 (11.3)	4 (6.2)	0.29
- Tobacco use	3 (4.2)	2 (3.1)	0.72
**Charlson index** (median ± IQR, min–max)	6.0 ± 4 (0–11)	5.0 ± 4 (0–11)	0.220
**Number of comorbidities per person** (median ± IQR, min–max)	3.0 ± 3 (0–7)	3.0 ± 3 (0–8)	0.39
**Hospital length of stay** (day, median ± IQR, min–max)	26 ± 25 (2–198)	31 ± 34 (2–133)	0.34
**Number of patients admitted to the ICU** (*n*, %)	41 (57.7)	34 (52.3)	0.52
**Proportion of sepsis** (*n*, %)	35 (49.3)	32 (49.2)	0.99
**30-day all-cause mortality** (*n*, %)	22 (31.0)	17 (26.2)	0.53
**1-year all-cause mortality** (*n*, %)	47 (66.2)	43 (66.2)	0.99

ICU: intensive care unit, IQR: interquartile range.

## Discussion

### Key findings of our study

The primary aim of our study was to compare the outcomes associated with bloodstream infections attributed to *E.* spp. in adult patients with and without active onco-hematological malignancies, in a national center over a 2.5-year long period. Our study revealed enterococcaemia as a clinical entity characterized by a pronounced mortality rate, with 45% of patients succumbing within the initial 30 days. Moreover, a substantial proportion of these fatalities occurred among individuals under intensive care suffering from active onco-hematological malignancies. Polymicrobial infections were identified in approximately a quarter of, and nosocomial origin was documented in nearly half of all cases. The incidence of confirmed infective endocarditis was notably low, potentially attributable to the limited utilization of cardiac imaging procedures. Intriguingly, the frequency of intensive care admissions was elevated (54.6%), a trend which has been potentially influenced, at least in part, by the prevailing SARS-CoV-2 (Severe Acute Respiratory Syndrome-Coronavirus-2) pandemic during the study period. Altough these findings are clinically relevant for all patients, we did not find statistically significant differences in clinical outcomes between our patient groups. The only baseline differences were in the proportion of cerebrovascular diseases (21.3 vs 5.8% *P* = 0.01), and the use of systemic glucocorticoids (0.0 vs 2.1% *P* = 0.04).

### Previous findings from the literature

Our main findings mostly align with global data, as documented in international literature. Enterococcal bloodstream infections consistently exhibit elevated in-hospital mortality rates [7, 13]. Predisposing factors, including chronic cardiovascular and renal diseases, as well as underlying active solid organ and hematological malignancies, have been identified as risk factors for the development of enterococcaemia [9, 14]. A recent Japanese study also revealed male gender as an additional risk factor for severe infections [15]. This can be partially reflected in our study, as 53.8% of deceased patients were males. Notably, nosocomial acquisitions constitute the predominant mode of infection among adult patients with a compromised immune status and a substantial comorbidity burden, which are risk factors for early mortality. While certain studies question the reported impact of enterococci on overall mortality, others identified a significant excess mortality of 31% in similarly vulnerable patients afflicted by *Enterococcus* spp. [16–19].

A particularly vulnerable group are adults with underlying active hematological malignancies, as they exhibit a susceptibility to strains demonstrating *in vitro* resistance to antibiotics due to prolonged hospitalizations and antimicrobial treatments, thereby amplifying the risk of in-hospital mortality [20–22]. A study conducted by Billington et al. found 11% increased relative risk for enterococcal bloodstream infections for any type of malignancies and 33.4% increased relative risk for hematological malignancies [9]. As the proportion of infections caused by multidrug-resistant Gram-positive bacteria in BSI was notably stable during recent years, the incidence and outcomes of infections with vancomycin-resistant enterococci (VRE) remain the main focus of interest in the literature [23]. In a study done in Pakistan, the authors found that even though VRE incidence decreased over their study period, the 30-day mortaity related to VRE infections increased to 44.8% [23].

A study conducted by Cho et al. showed that the *in vitro* antimicobial resistance profiles of *E. faecium* species were different among hematology patients, compared to non-hematology patients. The authors attributed the differences to the widespread use of fluoroquinolones, as these antibiotics are commonly administered as chemoprophylaxis for these patients [24]. Moreover, fecal VRE growth is known to be predisposed by chemotherapy and certain antibiotics, such as third-generation cephalosporins, vancomycin and metronidazole, also commonly administered among onco-hematology patients [25]. A heightened risk for BSIs is also accountable in the gastrointestinal colonization with *E. faecium*, as this step usually precedes BSI [25]. These factors alltogether predispose for poorer clinical outcomes, as patients with hematological diseases have significantly lower 30-day overall mortality due to VRE bloodstream infections, compared with non-VRE strains [26]. Finally, colonisation with non-faecalis and non-faecium enterococcal strains might also be more prevalent among immunocompromised cohorts [27].

We have two possible explanations for the lack of statistically significant differences between clinical outcomes of our study subgroups. A recent study about enterococcal bloodstream infections performed in Qatar documented higher mortality among patients with malignant diseases. As for patients with non-malignant diseases, chronic kidney disease resulted in increased mortality, while elderly male patients with diabetes mellitus and chronic kidney disease were at higher risk for acquiring enterococcal BSIs [28]. In our study, both subgroups exhibited a high proportion of elderly patients with diabetes mellitus and comorbidities. Other not shown comorbidities included mostly thyorid diseases which are not a known risk factors for severe enterococcal disease. In another study, coinfections were also associated with higher mortality, which is also a plausible explanation [29]. In a Japanese study, one of the main predictive factors for mortality was the Charlson Comorbidity Index (CCI), as a CCI of 3–4 had an adjusted odds ratio of 8.79, while a CCI ≥5 showed an adjusted odds ratio of 17.6 for mortality [30]. This may also partially explain why there was no statistically significant difference of mortality between subgroups, as both groups had a CCI ≥5. Another factor that may have impacted our results is the ongoing SARS-CoV-2 pandemic during the study period. The pandemic has ushered in an escalation of nosocomial infections, particularly bloodstream infections, with a marked surge of enterococcaemias [31, 32]. Recent studies, such as that conducted by Giacobbe et al., have calculated a 18 and 9% relative incidence of *E. faecalis* and *E. faecium* bloodstream infections among patients treated in ICUs for SARS-CoV-2, positioning them as the second and fourth most common pathogens, respectively [33]. Similarly, in Italy, the incidence of bloodstream infections caused by *Enterococcus* spp. demonstrated a high prevalence of 55.8% among adults requiring intensive care unit admission [34]. The COVID-19 pandemic also escalated the usage of systemic corticosteroids, immunomodulatory therapies and probably cephalosporins, and immunosuppression and inappropiate antibiotic therapies are documented risk factors for a fatal enterococcal BSI [35]. A higher number of severe comorbidities may also increase mortality in these infections [36]. We hypothesize that these circumstances may have contributed to the differences described in our study, as compared to international data.

Finally, several studies within the literature focused on the imperative of mitigating in-hospital mortality associated with bloodstream infections caused by *Enterococcus* spp. Timely and appropriate empirical antibiotic therapy, coupled with infectious disease consultations and the risk stratification of vulnerable patient groups, may emerge as pivotal measures to reduce early mortality [13, 37, 38].

### Limitations

While we feel that our study contributes valuable insights, its single-center and retrospective nature renders it susceptible to observational bias. Potential limitations also arise from the subjectivity shaping diagnostic and therapeutic decisions by attending physicians. Due to insufficient case numbers no comparison could be done in microbiological characteristics in our subgroups. Additionally, the relatively modest sample size and conceivable data heterogeneity may limit the generalisability of our findings.

## Summary

In conclusion, bloodstream infections caused by *E.* spp. showed high rates of short- and long-term mortality. Our study did not reveal differences in the long-term mortality of adult patients with and without active onco-hematological malignancies due to bloodstream infections caused by *E.* spp.

## Funding sources

BGSz received the János Bolyai Research Scholarship of the Hungarian Academy of Sciences (BO/00105/23/5) and a research grant from the “OTKA” Postdoctoral Excellence Programme 2023 of the National Research, Development and Innovation Office of Hungary (PD-147276). The article itself did not receive any external funding.

## Authors' contributions

BM: data collection, data analysis and preparation of the manuscript; BK: data collection, preparation of study protocol, process, isolation and characterisation of samples, AB: process, isolation and characterisation of samples LK: data collection, preparation of study protocol, process, isolation and characterisation of samples, KK: preparation of study protocol, process, isolation and characterisation of samples, JS: preparation of study protocol, BGSz: data analysis, preparation of study protocol, and preparation and review of the manuscript; BL: preparation of study protocol, review of the manuscript.

## Conflicts of interest

The authors declare that the research was conducted in the absence of any commercial or financial relationships that could be construed as a potential conflict of interest.

## Abbreviations

BSI: bloodstream infection
CRP: C-reactive protein
ICU: intensive care unit
LOS: length of stay
PCT: procalcitonin
SARS-CoV-2: severe acute respiratory syndrome coronavirus-2
TEE: transoesophageal echocardiography
TTE: transthoracic echocardiography
VRE: vancomycin-resistant enterococci
